# Biotechnological Applications Derived from Microorganisms of the Atacama Desert

**DOI:** 10.1155/2014/909312

**Published:** 2014-07-23

**Authors:** Armando Azua-Bustos, Carlos González-Silva

**Affiliations:** ^1^Blue Marble Space Institute of Science, Seattle, WA 98109, USA; ^2^Centro de Investigación del Medio Ambiente (CENIMA), Universidad Arturo Prat, 1110939 Iquique, Chile

## Abstract

The Atacama Desert in Chile is well known for being the driest and oldest desert on Earth. For these same reasons, it is also considered a good analog model of the planet Mars. Only a few decades ago, it was thought that this was a sterile place, but in the past years fascinating adaptations have been reported in the members of the three domains of life: low water availability, high UV radiation, high salinity, and other environmental stresses. However, the biotechnological applications derived from the basic understanding and characterization of these species, with the notable exception of copper bioleaching, are still in its infancy, thus offering an immense potential for future development.

## 1. Introduction

The Atacama Desert, located in northern Chile between latitudes 17° and 27° south, has average annual rains of less than 2 mm [1]. In comparison, other known deserts in the world, like the Mojave Desert in North America [2] or the Sahara Desert in Africa [3], have average annual rains of 116 mm and 100 mm, respectively. These extremely low rain rates have determined the Atacama Desert to be classified as a hyperarid desert [4] (a desert with an aridity index of less than 0.05, as the evapotranspiration of water from its soils is much higher than the inputs of rains). The Atacama Desert is also unique as it is believed to be the oldest desert on Earth, being arid for the last 150 million years and hyperarid for the past 15 million years [5, 6].

**Figure 1:**
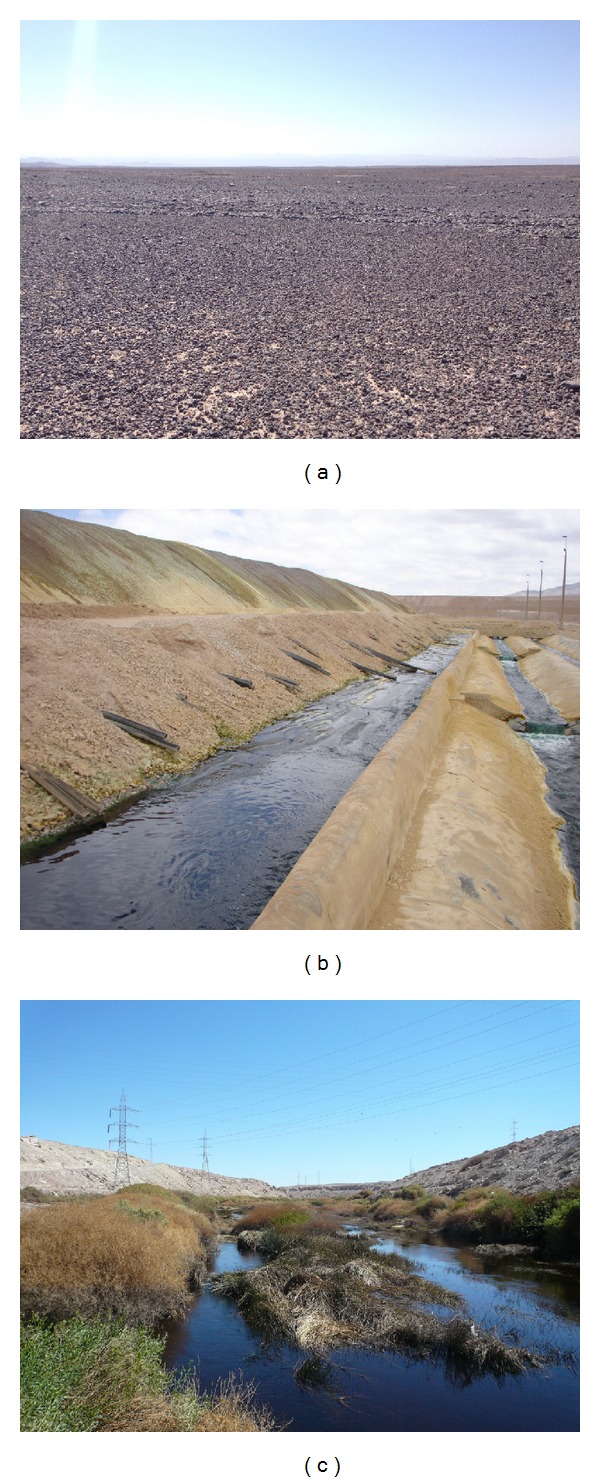
Examples of habitats of the Atacama Desert from where biotechnological applications have been derived or used. (a) The central valley, the hyperarid core of the Atacama Desert. (b) Heap bioleaching at Radomiro Tomic, an open pit copper mine owned by the Chilean Copper Corporation (Codelco). Note the copper rich blue-green solution obtained from the heaps. (c) The Loa River, a typical arsenic rich river of the Atacama Desert. Image credits: Panels A and C: Armando Azua-Bustos. Panel B: Armando Azua Aroz.

Thus, the Atacama has been an extremely dry desert for a very long time and only forty years ago it was thought that nothing could live in its seemingly barren landscapes (Figure 1(a)). However, during the past ten years, culture dependent and independent methods have unveiled a plethora of microorganisms (Bacteria, Archaea, and Eukarya) that were able to adapt and evolve in very specific and unexpected habitats of this desert [7]. Habitats as diverse as the underside of quartz rocks [8], fumaroles at the Andes Mountains [9], the inside of halite evaporites [10], and caves of the Coastal Range [11, 12] showed that microbial life found novel ways to adapt to the extreme conditions typical of the Atacama: extremely low water availability, intense solar radiation, and high salinity (for a more complete description of Atacama's microbial species, please see our recent review on this subject [7]). However, up to date, very few works have gone beyond the descriptive stage of establishing what types of microorganisms may be found in specific microenvironments [13], thus explaining the incipient biotechnological applications derived from knowledge that still being gained.

The study of the molecular strategies used by microbial life in other extreme environments (high temperature, for example) gave rise to many biotechnological applications that are now of standard use [14]. In a similar way, the characterization of the molecular strategies evolved by microorganisms of the Atacama to cope with its exceptional abiotic stresses (desiccation in particular) should be multiple and unique, and, thus, novel sources of metabolites and genes for the biotechnological industry. In this review, the few reported cases of the biotechnological use of Atacama Desert microorganisms to date are summarized.

## 2. Applications Derived from Members of the Bacteria Domain

*Copper Bioleaching.* Copper bioleaching or “biomining” allowed the usage of insoluble copper sulphides and oxides through hydrometallurgy, as opposed to the traditional technology of pyrometallurgy. Compared to pyrometallurgy, bioleaching has the advantage of being a simpler process, requiring less energy and equipments (Figure 1(b)). In addition, bioleaching does not produce sulfur dioxide emissions, an important factor for the Chilean mining towns which were usually built alongside the extracting operations in Chile (most of which are located in the Atacama Desert). Bioleaching also offered a better treatment of low grade (again the usual case in Atacama copper ores) or waste ores and in many cases it is the only way to treat them. Low-grade ores (0.6% and less) are abundant in Chile, but their processing by pyrometallurgy in most cases is not economical. Through bioleaching, copper was able to be extracted from ore minerals like chalcopyrite (CuFeS2), with the crucial contribution of chemolithotrophic microbial species extremely tolerant to low pH, which use the reduced sulphur as an energy source. The most known of these microorganisms is* Acidithiobacillus ferrooxidans* [15], but other species, like* Leptospirillum ferrooxidans*,* Sulfobacillus acidophilus,* and* Acidimicrobium ferrooxidans,* are thought to also participate in the bioleaching process [16, 17].

In Chile, the first mine that introduced bioleaching was Sociedad Minera Pudahuel (a copper mine not located in the Atacama) in the 1980s. Today, this process is extensively used in the Chilean copper mining industry [18, 19], reaching over 1.6 million tons of copper per year [19]. It has been estimated that Chile's copper actual reserves would increase up to 50% if all copper sulphides could be economically treated by bioleaching [19].

*Acidithiobacillus ferrooxidans* has been identified in different places of the Atacama Desert [20, 21]. Some of these species have been found in sulfidic mine tailings dumps in the marine shore at Chañaral Bay [21], located at the Coastal Range of the Atacama. This is of particular interest as these species were found to be halotolerant iron oxidizers, active at NaCl concentrations up to 1 M in enrichment cultures. High concentrations of chloride ions inhibit the growth of the acidophilic microorganisms traditionally used in biomining [22]. Thus, the finding of halotolerant bioleaching species would allow the use of seawater for biomining operations in the future, a very important advancement in a region, where water availability has always been extremely low.

Bioleaching strains found in the Atacama Desert have been recently patented, as is the case of* Acidithiobacillus ferrooxidans* strain Wenelen DSM 16786 [23] and* Acidithiobacillus thiooxidans* strain Licanantay DSM 17318 [24] (US Patent numbers 7,601,530 and 7,700,343). Both strains showed improved oxidizing activity when compared to standard strains isolated elsewhere, like* Acidithiobacillus ferrooxidans* ATCC 23270 and* Acidithiobacillus thiooxidans* ATCC 8085. Strain Wenelen, an iron and sulfur oxidizing microorganism, was particularly efficient in oxidizing chalcopyrite, while strain Licanantay, a strict sulfur oxidizer, showed activity in both primary and secondary sulfured minerals, such as chalcopyrite, covellite, bornite, chalcocite, enargite, and tennantite [24–26].

Recently, the comparative genomic analysis and metabolomic profiles of these two strains were obtained, which turned helpful for determining basic aspects of its regulatory pathways and functional networks, biofilm formation, energy control, and detoxification responses [27, 28].

As for Archaea, although some species have been reported in acid mine drainage in the Atacama [21], there are yet no reports of strains specifically isolated for industrial use.

*Biomedicine.* Soils of the Atacama shelter a number of bacterial species with promising characteristics for the biomedical industry. One of the first descriptions of microorganisms that are known to produce such biomolecules was published in 1966 by Cameron et al. [29]. Commissioned by NASA, this group approached the Atacama as a way to obtain basic information on terrestrial desert environments and its microbiota in order to develop and test the instruments to be taken to Mars ten years later by the Viking Mission. Among others, they were among the first to report the presence of* Streptomyces* species,* Bacillus subtilis aterrimus*,* Bacillus brevis*,* Bacillus cereus,* and* Micrococcus caseolyticus*; however, no details of biomolecules produced by these species were later reported.

Almost forty years passed until a groundbreaking report by McKay's group in 2003 [30] showed that when the experiments performed by the Viking landers on the surface of Mars were repeated with soils of the Yungay region of the Atacama Desert, the same results were obtained essentially. This leads to the recognition of the Atacama Desert as one of the driest places on Earth, causing then a surge of reports focusing on the characteristics of various microenvironmental conditions in the Atacama and its related microbiology [7].

Later on, the interest in the potential biomedical use of these recently reported species started, focusing on species of the Actinobacteria, as these were previously known as synthesizers of useful molecules [31]. Among this latter class, species like* Amycolatopsis*,* Lechevalieria,* and* Streptomyces* have been reported at various arid and hyperarid sites of the Atacama [32].

Members of the* Streptomycetes* are a well-known source of antibiotics [33], and* Lechevalieria* species are known to have nonribosomal peptide synthase (NRPS) gene clusters that synthesize antitumoral compounds [31]. Accordingly, of the species found by Okoro's group [32], all of the* Amycolatopsis* and* Lechevalieria* and most of the* Streptomyces* isolates tested positive for the presence of NRPS genes. This same group determined later the metabolic profile of one of these* Streptomyces* strains (strain C34), identifying three new compounds from the macrolactone polyketides class [34] and other compounds like deferoxamine E, hygromycin A, and 5′′-dihydrohygromycin. These compounds showed a strong activity against the Gram-positive bacteria tested (*Staphylococcus aureus*,* Listeria monocytogenes,* and* Bacillus subtilis*), but weak activity against the tested Gram-negative bacteria (*E. coli* and* Vibrio parahaemolyticus*).

In a parallel report, they also found that strain C34 synthetized four new antibiotics of the ansamycin-type polyketides with antibacterial activity against both* Staphylococcus aureus* ATCC 25923 and* Escherichia coli* ATCC 25922 [35]. In particular, chaxamycin D4 showed a selective antibacterial activity against* S. aureus* ATCC 25923. Another of these strains,* Streptomyces* sp. C38, synthetized three new macrolactone antibiotics (atacamycins A–C) which exhibited moderate inhibitory activity against the enzyme phosphodiesterase (PDE-4B2) [36]. Inhibitors of PDE (the most famous of this group being Viagra) can prolong or enhance the effects of physiological responses mediated by cAMP and cGMP by inhibition of their degradation by PDE and are considered potential therapeutics for pulmonary arterial hypertension, coronary heart disease, dementia, depression, and schizophrenia [37]. In the case of atacamycin A, it also showed anti proliferative activity against cell lines of colon cancer (CXF DiFi), breast cancer (MAXF 401NL), uterus cancer (UXF 1138L), and colon RKO cells [36].

Similar positive results were obtained by Leirós et al., 2014 [38] in which seven molecules synthetized by* Streptomyces* sp. Lt 005, Atacama* Streptomyces C1,* and* Streptomyces* sp. CBS 198.65 were tested against hydrogen peroxide stress in primary cortical neurons as potentially new drugs for the avoidance of neurodegenerative disorders such as Parkinson's and Alzheimer's diseases. The reported compounds inhibited neuronal cytotoxicity and reduced reactive oxygen species (ROS) release after 12 h of treatment. Among these compounds, the quinone anhydroexfoliamycin and the red pyrrole-type pigment undecylprodigiosin showed the best protection against oxidative stress with mitochondrial function improvement, ROS production inhibition, and increase of antioxidant enzymes like glutathione and catalase. In addition, both compounds showed a modest caspase-3 activity induced by the apoptotic enhancer staurosporine.

In a different work, another group of secondary metabolites, called abenquines, were found to be synthetized by* Streptomyces* sp. Strain DB634, isolated from the soils of the Altiplano of the Atacama [39]. These abenquines (A–D) showed modest inhibitory activity against* Bacillus subtilis*, dermatophytic fungi, phosphodiesterase type 4b, and antifibroblast proliferation (NIH-3T3).

An interesting case to discuss in this section of a commercially successful, but controversial, example of a compound produced from an Actinobacteria isolated from another well-known Chilean environment is that of rapamycin (also known as sirolimus), isolated by Brazilian researchers from a strain of* Streptomyces hygroscopicus* endemic of Eastern Island, or* Rapa Nui* [40]. Rapamycin was originally used as an antibiotic, but later on it was discovered to show potent immunosuppressive and antiproliferative properties [41, 42] and even claimed to extend life span [43]. Sadly, nothing of this development benefited the Chilean economy, as agreements like the United Nations Rio Declaration on Environment and Development were yet to be established.

*Arsenic Bioremediation.* Conventional arsenic removal in drinking water such as reverse osmosis and nanofiltration are effective and able to remove up to 95% of the initial arsenic concentrations, but the operating costs of these plants are high [44]. In addition, the oxidation of As (III) to As (V) is a prerequisite for all conventional treatment processes, and as this is an extremely slow reaction toxic and costly oxidants such as chlorine, hydrogen peroxide, or ozone must be used as catalysts [44, 45]. Thus, an attractive alternative solution for arsenic removal is bioremediation, as a wide variety of bacteria can use it as an electron donor for autotrophic growth or as an electron acceptor for anaerobic respiration [46–48].

In the case of the Atacama Desert, the first steps leading to the biosequestration of arsenic by endemic microorganisms are now being taken. This toxic metalloid is naturally found in rivers of the Atacama Desert (Figure 1(b)) as arsenate As (V) and the most toxic species arsenite As (III) [49–51]. Among other negative biological effects, arsenate, being a chemical analog of phosphate, inhibits oxidative phosphorylation and arsenite binds to sulfhydryl groups of proteins [52]. It is precisely in the sediments of one of these rivers, (Camarones river near the coastal city of Arica) with arsenic concentration, in water (1100 *μ*g L^−1^) and sediments (550 *μ*g L^−1^) that 49 isolates were identified and distributed between the *α*-Proteobacteria (5 isolates), *β*-Proteobacteria (13 isolates), and *γ*-Proteobacteria (26 isolates) [44, 53]. Most of these species belonged to the genera* Alcaligenes*,* Burkholderia*,* Comamonas, Enterobacter, Erwinia*,* Moraxella*,* Pantoea*,* Serratia*,* Sphingomonas*, and* Pseudomonas* [53], of which* Alcaligenes*,* Burkholderia*,* Sphingomonas, Pantoea*,* Erwinia*, and* Serratia* were not previously reported in literature as arsenic tolerant. Fittingly, eleven of the arsenic-tolerant isolates had the gene* ars* that codes for the critical enzyme involved in this reaction, arsenate reductase [53]. In a later work from this group, it was found that one of the species isolated,* Pseudomonas arsenicoxydans* strain VC-1, was able to tolerate up to 5 mM of As (III), being also capable of oxidizing at high rates the totality of the arsenite present in the medium, with lactate as a carbon source [54]. Thus, the characterization of these species in experimental bioreactors will certainly offer interesting options for future water and soil bioremediation [55].

## 3. Applications Derived from Microbial Members of the Eukarya Domain

*Biomedicine.* Carotenoids are lipid soluble tetraterpenoid pigments synthesized as hydrocarbons (carotene, e.g., lycopene, *α*-carotene, and *β*-carotene) or their oxygenated derivatives (xanthophylls, e.g., lutein, *α*-cryptoxanthin, zeaxanthin, etc.) by microorganisms and plants [56]. In these organisms, they play multiple and critical roles in photosynthesis, by maintaining the structure and function of photosynthetic complexes, contributing to light harvesting, quenching chlorophyll triplet states, scavenging reactive oxygen species, and dissipating excess energy [57, 58]. Up to date, more than 700 carotenoids have been described [59]. Yellow, orange, and red carotenoids are used as pharmaceuticals, animal feed additives, and colorants in cosmetics and foods. Interest in dietary carotenoids has increased in the past years due to their antioxidant and anti-inflammatory potential [60, 61], as they are very efficient quenchers of singlet oxygen and scavengers of other reactive oxygen species [62]. Carotenoids are also important precursors of retinol (vitamin A) [62, 63].

Among other sources, species of the halophilic biflagellate unicellular green alga* Dunaliella* (Chlorophyta), like* Dunaliella salina,* are industrially cultivated as a natural source of beta-carotene around the world, including Chile [64]. Under conditions of abiotic stress (high salinity, high temperature, high light intensity, and nitrogen limitation) up to 12% of the algal dry weight is *β*-carotene [65, 66] which accumulates in oil globules in the interthylakoid spaces of their chloroplast [65]. In addition, as a defense mechanism against hypersalinity,* D. salina* synthetizes high amounts of the compatible solute glycerol, another molecule of economic value [67].

There are several species of the genus* Dunaliella* reported in the Atacama Desert, mainly in hypersaline lagoons [68, 69] and even growing aerophytically on cave walls [11]. In the case of* D. Salina* Isolate Conc-007, isolated from the Salar de Atacama, it was found to be capable of synthesizing 100 pg of beta-carotene per cell, two to four times higher than other species used in commercial beta-carotene production [69, 70]. In turn,* D. salina* SA32007, also isolated from the Salar de Atacama, synthesized triglycerides-enriched lipids under nitrogen deficiency conditions, a potentially relevant result for biodiesel production [71]. Important differences in the carotenogenic capacity of the* D. salina* strains have been shown to be dependent on the high genetic diversity of member of this species [69]. This is highly relevant for the case of the Chilean species, as it seems that they may be better producers of *β*-carotene and other biomolecules in comparison to other species of the world.

An additional factor to consider in this case is that* Dunaliella* production facilities elsewhere (Australia, China, and India) are located in areas where solar irradiance is maximal, climate is warm, and hypersaline water is available [57], which are precisely the characteristics of most areas of the Atacama; thus, growth facilities may well be developed in this desert using endemic strains. In addition, adaptive laboratory evolution [72] and metabolic engineering may be applied to the Atacama species in the future, as these methods have recently been investigated and accomplished [72, 73].

## 4. Final Comments

The brevity of this review reflects how little has been advanced to date in the biotechnological use of members of the microbial world found in the Atacama Desert. This may be understood. Although the Atacama is well known for its extreme dryness, up to 2003, there was little interest in exploring and characterizing its potential microbial ecosystems, as it was generally supposed to be sterile. Ten years later, microbial life has been found in most if not all of its habitats, from high thermal springs on the Andes Mountains to caves of the Coastal Range, thus building a yet ongoing descriptive stage of extant microbial ecosystems. Therefore, it is not surprising that the technological stage of research is just beginning.

As previously mentioned, the Atacama Desert is unique as it has been the most arid place on Earth for a very long time, imposing the same selection pressure over the life forms that arrived and then coevolved with it. Fittingly, all reports to date have shown that these species are unique in the way the capture, store and use water, tolerate solar radiation, high saline conditions and low soil nutrients [7, 74]. Thus, with the exception of copper bioleaching, now a multimillion dollar industry, we believe there is an immense biotechnological potential waiting to be discovered and developed in relation to the tolerance to the aforementioned abiotic stresses.

Most groups now are still reporting various degrees of tolerance to abiotic stresses in the frame of basic research (extreme environments and astrobiology in particular), and we foresee that during the next years the detailed understanding of the physiological and molecular mechanisms involved in the many abiotic stress tolerances shown by these species should increase to a great extent (see [13], e.g.). “Omics” techniques, like genomics, proteomics, and metabolomics, and high-throughput technologies will be key in elucidating processes and mechanisms involved in these tolerances and then in identifying and characterizing key molecules of potential use.

In the case of bioleaching, we envision that new bacterial and archaeal strains will appear in the market. Tailor-made combinations of mine-specific strains will probably be isolated, characterized, and patented in order to maximize the dissolution of copper from the particular complexity of minerals characteristic of each place. In addition, other properties of the mine may be taken into account in the determination of the characteristics of the strain mixture, like water quality, soil temperature, and so forth.

In the case of biomolecules of interest for the biomedical industry, there are already a handful of groups characterizing potentially interesting biomolecules from bacterial strains isolated from the hyperarid areas and hypersaline lagoons of the Atacama Desert, biomolecules which have just been identified, and their activities preliminary tested. These isolates are few and are representatives of a very small fraction of the habitats of the Atacama, so novel strains and metabolites will certainly appear in the near future. Microorganisms of the dry core of the Atacama will be of particular interest, as we expect that these species, being subjected to the most extreme conditions, should produce a number of biomolecules involved in the competition for scarce resources.

With the increasing pressure of finding new drugs able to handle antibiotic-resistant pathogens [75], extreme environments are now being investigated in detail [76], and the Atacama Desert, given its unique peculiarities, may be a prime place to explore.
